# Risk Stratification for Sudden Cardiac Death in Non-Ischaemic Dilated Cardiomyopathy

**DOI:** 10.1007/s11886-019-1236-3

**Published:** 2019-11-25

**Authors:** M. Akhtar, P. M. Elliott

**Affiliations:** 1 Cardiology clinical research fellow, University College London, Institute of Cardiovascular Science, Paul O’Gorman building, 72 Huntley street, London, WC1E 6AG UK; 2 Chair of Cardiovascular Medicine, University College London, Institute of Cardiovascular Science, Paul O’Gorman building, 72 Huntley street, London, WC1E 6AG UK

**Keywords:** Dilated cardiomyopathy, Sudden cardiac death, Genetics, Implantable cardioverter-defibrillator

## Abstract

**Purpose of Review:**

Non-ischaemic dilated cardiomyopathy (DCM) occurs in 1 in 2500 individuals in the general population and is associated with considerable morbidity and mortality. Studies involving large numbers of unselected DCM patients have led to consensus guidelines recommending implantable cardioverter-defibrillator (ICD) implantation for protection against sudden cardiac death (SCD) in those with LVEF ≤35%. The purpose of this article is to review the literature for other potential markers including serological, electrocardiographic, echocardiographic, cardiac magnetic resonance, ambulatory ECG and genetic data, to highlight other potential markers that may optimise risk stratification for SCD in this cohort and thereby allow a more personalized approach to ICD-implantation.

**Recent Findings:**

Recent studies including the Danish study to assess the efficacy of ICDs in patients with non-ischemic systolic heart failure on mortality (DANISH) trial have questioned the benefits of ICD implantation in this group of patients with no changes in all-cause mortality. Recent pooled cohorts of patients with genetic DCM and in particular in those with Lamin A/C (LMNA) mutations have identified patients at increased risk of SCD and allowed the creation of algorithms to prognosticate SCD risk in mutation carriers. Furthermore, genetic testing has identified other DCM-causing genes including filamin C (*FLNC*) and *RBM20* which may be associated with higher rates of ventricular arrhythmia.

**Summary:**

To date, risk-stratification for SCD has been hampered by the utilisation of heterogenous subsets of idiopathic DCM patients and by use of static risk models where predictions are based on a single time point with a lack of consideration of disease progression. The current focus of personalised risk-stratification for SCD is shifting towards better characterisation of underlying DCM aetiology and the development of multi-parametric risk-stratification models that incorporate time-dependent disease characteristics and novel biomarkers.

## Introduction

Non-ischaemic dilated cardiomyopathy (DCM) occurs in 1 in 2500 individuals in the general population [1•] and predisposes to end-stage heart failure (ESHF) and malignant ventricular arrhythmia (VA). The annual incidence of sudden cardiac death (SCD) in DCM is 2-4% with sudden death accounting for up to half of all deaths [2, 3]. This is also reflected in registry data of survivors of an aborted cardiac arrest, where DCM is the underlying aetiology in 10-19% [4]. Importantly, SCD may be the initial manifestation of DCM in previously asymptomatic individuals.

While most data suggest that implantable cardioverter defibrillators (ICD) prevent SCD and lower mortality in DCM cohorts by up to 31% [5], only 20-25% of patients with primary prevention ICD receive an appropriate shock within 5 years of device implantation highlighting that current risk stratification algorithms remain suboptimal [6, 7]. Most studies examining predictors of SCD in DCM populations have focussed on left ventricular ejection fraction (LVEF) as a stratifier. However, whilst it is true that SCD events are relatively numerous in those with severe LV systolic impairment, the proportion of SCDs compared to other causes of cardiovascular mortality such as ESHF is much greater in individuals with milder reductions in LVEF. The resultant paradox is that many patients presenting with SCD have a LVEF that does not meet consensus criteria for primary prevention ICD implantation and often in those without preceding symptoms of heart failure [8]. Moreover, a third of adverse events can occur late (>72 months) post symptom onset, despite optimal medical therapy (OMT) [9].

Given these limitations of LVEF as a solitary risk marker in DCM, alternative biochemical, clinical and imaging risk markers have been sought. As yet, most studies are based on retrospective or observational registry data, but a number of potential candidates for risk predictors are emerging and discussed below.

### Serological markers

Troponin T (TnT) and NT-proBNP (BNP) are serum biomarkers that indicate the severity of the heart failure phenotype and/or myocardial injury and are correlated with prognosis [10, 11]. Raised BNP levels have been shown to predict SCD risk with a relative risk of 3.7 [12] and in patients with ICDs, raised BNP levels are associated with an over two-fold increase in the risk of VA [12, 13].

C-reactive protein (CRP) is a marker of inflammation, and significantly higher levels have been shown to be present in DCM patients dying within 5 years of hospitalization compared to survivors [14]. In those with acute heart failure necessitating hospitalization, CRP levels are also associated with an increased likelihood of intensive care admission and in-hospital mortality [15]. Elevated high-sensitivity CRP (hs-CRP) levels are associated with all-cause mortality and rehospitalization in DCM although not with SCD specifically [16].

Serum levels of pro-inflammatory cytokines, including IL-1, are significantly increased in patients with heart failure [17, 18]. Interleukin 1-B has been demonstrated to affect cardiac remodelling and contributes to post-inflammatory myocardial fibrosis and DCM in animal models [19]. Increasing IL-1 levels are associated with progressive severities of heart failure and atrial fibrillation [20]. ST2, another cytokine, has protective actions against myocyte fibrosis and soluble ST2 receptor is released in response to myocardial stress [18]. An increase in soluble ST2 levels has been demonstrated to be an independent predictor of one-year mortality in acute decompensated heart failure [21] and of hospitalizations and mortality in chronic heart failure. However, its usage in prognostic models did not demonstrate a significantly improved means of risk reclassification [22]. Currently, evidence to demonstrate the routine use of cytokine levels for SCD prognostication is lacking.

### Electrocardiographic markers

Several studies have analysed the association of various electrocardiographic markers with the risk of SCD in DCM including QRS duration, QRS fragmentation, microvolt T-wave alternans and QRS-T angle. In a detailed meta-analysis of markers of SCD, both depolarization and repolarization ECG abnormalities were more common in DCM patients with SCD. Fragmented QRS complexes, T-wave alternans, abnormal signal-averaged ECG (SAECG) and prolonged QRS duration were all significantly associated with SCD with decreasing magnitudes of relative risk [23].

QRS duration is a relatively easy, reproducible marker and has been shown to be a significant predictor of cardiovascular mortality, SCD and appropriate ICD shock in patients with DCM [24]. In a study of 710 DCM patients, QRS duration at baseline evaluation was independently associated with arrhythmic endpoints (SCD, aborted SCD, appropriate ICD therapy or sustained VT) at one-year [9]. Similar conclusions were identified in sub-group analysis of the SCD-HeFT cohort where patients with QRS≥120ms had greater survival benefit with ICD compared to placebo therapy although this was not stratified according to ischaemic or non-ischaemic cardiomyopathy subtypes [7]. Other studies have utilised a combination of ECG and imaging predictors to optimise risk stratification. For example, in a study of 531 patients with idiopathic DCM, a combination of broad QRS (QRS duration ≥120ms) together with late gadolinium enhancement (LGE) on cardiac magnetic resonance (CMR) provided incremental benefit to LGE alone in predicting all-cause mortality (HR 4.3; 95%CI 1.2-15.5; p=0.03) [25]. QRS fragmentation is also more common in patients with chronic heart failure and SCD [26] and is associated with all-cause mortality and arrhythmic events, independent of QRS duration [26, 27].

Microvolt T-wave alternans (TWA) is defined as a beat-to-beat alternation of T wave amplitude and reflects spatiotemporal repolarization heterogeneity which acts as a substrate for arrhythmia. TWA was initially thought to be a promising independent marker of SCD in patients with DCM [28–31], but conflicting data including a negative prospective substudy analysis of SCD-HeFT trial involving 490 patient have dampened enthusiasm for its use as an important electrocardiographic marker [32, 33].

### Ambulatory ECG Monitoring

24-hour Holter monitoring demonstrates non-sustained ventricular tachycardia (NSVT) in 40-60% of patients and polymorphic ventricular ectopy (VE) in up to 90% of DCM patients [34]. Data from electro-anatomical mapping catheter studies suggest that up to 80% of these ventricular arrhythmias are secondary to scar-related re-entry into the myocardium [35, 36]. However, in a study of 319 idiopathic DCM patients on OMT, NSVT was not found to be predictive of malignant VA (SCD, Sustained VT, ventricular fibrillation (VF), appropriate ICD shock) in patients with LVEF ≤35%. In those with LVEF >35%, however, the number of runs and length of NSVT were predictive of VA (HR 5.3, 95%CI: 1.6-17.9) [37]. Other studies of patients with moderate-severe heart failure failed to demonstrate that NSVT provided any incremental benefit in predicting patients at risk of SCD [38].

Sustained ventricular tachycardia (VT) occurs in under 5% of DCM patients and is a risk factor for SCD. Inducibility of VT on electrophysiology study (EPS) has been suggested as valuable for risk stratification in previous consensus guidelines, but most data suggest that EPS discriminate poorly between high and low-risk patients with respect to SCD. In the MADIT II study, inducibility of ventricular arrhythmia on EPS was associated with an increased likelihood of VT but not of VF and was not a good predictor of a composite endpoint of VT/VF [39, 40]. In current clinical practice, EPS is not routinely used to guide decision making on ICD implantation.

### Echocardiographic Risk Markers

Echocardiography is the most common imaging modality used to assess the severity of LVEF and thereby provide prognostic data in patients with DCM. Other echocardiographic parameters have also been considered including LV dimension, atrial size and in a study of 710 DCM patients, mitral regurgitation on baseline TTE was an important predictor of VA [9].

Newer echocardiographic technologies such a speckle tracking, have been suggested to provide incremental improvements in SCD stratification. For example, global longitudinal strain (GLS) is a marker of myocardial regional contractility and an impaired GLS may reflect myocardial fibrosis [41]. GLS can also identify subtle motion abnormalities in the ventricle prior to more overt changes or remodelling resulting in impairment in LVEF. Studies have demonstrated that impaired GLS is associated with increased arrhythmic events (sustained VT and cardiac arrest) (HR 1.3; 95%CI 1.1-1.5; p=0.01) and may be more predictive for arrhythmic events than LVEF [42].

Mechanical dispersion is the standard deviation between different myocardial segments of the time to peak negative strain. In a small study of 94 patients with DCM, mechanical dispersion was higher in patients that experienced arrhythmic events compared to those that didn’t (98±43ms vs 56±18ms) and was an independent predictor of arrhythmias (HR 1.28; 95%CI 1.1-1.5) [42].

### Cardiac Magnetic Resonance (CMR) Markers

CMR provides a reproducible assessment of LVEF and LV volumes, and with the administration of gadolinium contrast, data on myocardial scarring. T1 and T2 sequences are also used to quantify interstitial fibrosis and myocardial oedema.

Late gadolinium enhancement (LGE) is present in up to 38% of DCM patients [43]. Myocardial fibrosis can occur due to collagen accumulation resulting in interstitial expansion without myocardial necrosis (interstitial fibrosis) and from cardiomyocyte death (replacement fibrosis) [44]. Myocardial fibrosis is a substrate for VA in ischaemic heart disease where the scar represents a transition point between normal myocardium and fibrotic tissue resulting in the creation of slow-conduction re-entry circuits and ‘scar-related’ VT. This mechanism may also contribute to VT in some patients with DCM [45].

Several studies have demonstrated an association between fibrosis and SCD in patients with DCM [43, 46–50]. In the largest meta-analysis, incorporating 2948 DCM patients, LGE was significantly associated with an arrhythmic endpoint (OR 4.3, p<0.001) [51]. An arrhythmic endpoint occurred in 21% of patients with LGE (annual event rate of 6.9%) compared to 4.7% of patients (annual event rate of 1.6%) without LGE. Importantly, in the sub-category of patients with DCM and LVEF>35%, LGE was demonstrated to be significantly associated with an arrhythmic endpoint (OR 5.2, 95%CI: 3.4-7.9; p<0.001) [51].

Despite these extensive data, there are no randomised controlled trial (RCT) data to confirm the impact of these findings with respect to LGE and there is no conclusive evidence to demonstrate that patients with severe LVSD and without fibrosis are not at-risk of SCD. Other issues include a lack of standardization in the methodology, quantification and analysis of LGE as well as a lack of appreciable threshold of fibrosis over which arrhythmic risk elevates [52].

Some patients with DCM undergo endomyocardial biopsy (EMB) particularly if there are concerns of inflammatory conditions such as active myocarditis. EMB data may be utilised to provide data on the quantification of interstitial myocardial fibrosis in patients with DCM. This however is limited by procedural risks of EMB as well as by sampling error which may be particularly relevant with non-homogenous distributions of fibrosis. Native pre-contrast T1 times and extra-cellular volume fractions on cardiac magnetic resonance may reflect the degree of interstitial fibrosis in patients with DCM and offer a non-invasive method to quantify myocardial collagen composition [50, 53, 54]. In a multicentre prospective observational study involving 637 patients with DCM, T1-mapping indices were associated with all-cause mortality (p<0.001) [55]. Native T1 remained an independent predictor of all-cause mortality and of heart failure endpoints (hospitalization or ESHF-mortality) (HR 1.1; 95%CI: 1.1-1.2, p<0.001) [55]. However, this study did not analyse arrhythmic endpoints including SCD. In another single-centre study of 130 patients with ICDs and a mixture of ischaemic and non-ischaemic cardiomyopathy, native T1 value was an independent predictor of an arrhythmic endpoint consisting of appropriate ICD therapy or sustained VA (HR 1.1; 95%CI: 1.0-1.2) [56]. Further data is needed in unselected DCM cohorts to conclusively demonstrate the value of this modality in risk-stratification.

### Autonomic Dysfunction

Patients with heart failure often have altered sympathetic activation which predisposes to progressive ventricular dysfunction and arrhythmias and is likely to contribute to an increased risk of SCD [57]. Myocardial uptake of 123-metaiodobenzylguanidine (MIBG), a nuclear tracer with the same uptake and storage as norepinephrine, is reduced in heart failure and cardiac MIBG washout rate has been associated with SCD in patients with mild or moderate heart failure with LVEF <40% [58–61]. However, in a meta-analysis of various predictors of SCD in DCM, autonomic variables such as heart rate variability, heart rate turbulence and baroreflex sensitivity were not demonstrated to be significantly associated with SCD [23].

### Genetic Markers

A family history of sudden cardiac death is associated with SCD events in heart failure patients [62] and more than 1 in 4 patients with DCM carry a pathogenic genetic mutation. In a recent study of 487 DCM patients undergoing genetic testing, there was a trend towards increased malignant ventricular arrhythmia (SCD / VT / VF) in gene variant carriers (p=0.06) and increased heart-failure events (destination left ventricular assist device, heart transplantation or ESHF-mortality) [63]. This study demonstrated that LMNA or desmosomal mutation-carriers were at greatest risk of VA regardless of LVEF [63].

The number of disease-causing genes is considerable, but emerging data suggest that truncating mutations in titin (TTNtv) are the commonest cause of genetic DCM, accounting for between 15-25% of cases [64]. Patients with TTNtv appear to have high rates of positive remodelling on OMT and similar outcomes to patients without *TTN* mutations [65, 66]. Arrhythmic deaths do occur in *TTN* mutation carriers, but progressive heart failure predominates. In contrast, other disease genes appear to be associated with a much higher risk of SCD. Specific examples include the following:

### Lamin AC (LMNA)

*LMNA* mutations account for as many as 10% of cases of genetic DCM and are associated with early atrial and ventricular arrhythmia, premature conduction disease, SCD and progression to ESHF resulting in death or heart transplantation (HTx) [67–69]. *LMNA* mutations are associated with a high clinical penetrance by the age of 60 years and with considerable morbidity and mortality [70]. In a study of 269 *LMNA* mutation carriers followed up for a median of 43 months, 18% experienced malignant VA (4% an aborted SCD event, 9% an appropriate ICD therapy and over 4% had a SCD). This study identified the NSVT, male sex, non-missense mutations and LVEF <45% as risk factors for malignant VA [71]. A more contemporary study involving 839 *LMNA* mutation carriers identified the same risk factors but also found 1^st^ degree or higher AV block as a predictor [72]. This study and others highlight that conventional EF-threshold based guidelines for ICD implantation are inappropriate in *LMNA* mutation carriers and that higher EF thresholds should be accepted for ICD implantation.

### Filamin C (FLNC)

Truncating mutations in *FLNC* account for up to 4% of DCM cases [73] and are associated with frequent ventricular arrhythmia (82%). In one study, 15% of patients with *FLNC* mutations had a cardiac arrest with a mean age of 42±16 years and a mean LVEF of 39.6±12% [73]. Moreover, 20% of patients with a primary prevention ICD had an appropriate ICD shock, much higher than that seen in unselected DCM populations [73]. Although larger data sets with longitudinal follow-up are needed, preliminary data suggests that *FLNC* mutations are associated with a significant risk of VA events and lower thresholds for ICD implantation may need to be considered in this group.

### RNA-binding motif protein 20 (RBM20)

The RBM20 splicing factor is involved in the splicing of Titin (*TTN*) and calcium/calmodulin dependent kinase II delta (CAMK2D). A recent study of carriers of *RBM20* mutations identified an association with an arrhythmogenic DCM with increased incidence of VA [74]. 44% of carriers of *RBM20* mutations had sustained VA compared to 5% of *TTN* mutation carriers despite similar LVEF. In another multicentre study of 74 *RBM20* mutation carriers, there was a considerable family history of SCD (51%) and patients with *RBM20* mutations were more likely to have NSVT (43% vs 11%) and sustained VT (25% vs 2%) than idiopathic DCM cohorts [75].

### Phospholamban (PLN)

Mutations in the phospholamban gene are associated with an arrhythmogenic DCM characterised by low voltage ECG complexes and frequent ventricular arrhythmic events. The best characterised *PLN* variant is R14del which is a founder mutation in the Netherlands that accounts for 15% of DCM regionally. DCM patients with R14del are more likely to have an appropriate ICD shock (47% vs 10%) and heart transplantation (18% vs 2%) compared to those that do not carry a *PLN* mutation. R14del carriers are also more likely to have a family history of SCD at <50 years (36% vs 16%) with a mean age of SCD at 37.7 years; SCD often being the index presentation at a young age [76].

### Desmoplakin (DSP)

Mutations in the *DSP* gene are associated with ARVC but recent data suggest that they more typically cause a left-dominant arrhythmogenic cardiomyopathy. Variants in *DSP* are associated with left ventricular systolic dysfunction, myocardial fibrosis and ventricular arrhythmia [77]. Importantly, carriers of *DSP* mutations can develop sustained VT in the absence of severe LVSD and primary prevention ICD implantation is often considered in the presence of an abnormal LVEF [77].

### Sodium Voltage-Gated Channel Alpha Subunit 5 (SCN5A)

Mutations in *SCN5A* are associated with conduction disease and Brugada syndrome. Some mutations, including R222Q are associated with DCM, atrial arrhythmia, frequent premature ventricular complexes and SCD [78, 79]. Arrhythmias are reported in over 90% of *SCN5A* DCM cases [80]. There are reports of successful arrhythmia and ectopy treatment with quinidine in R222Q mutation carriers with improvement in LVEF [81].

### Conclusions: Implications for Primary Prevention ICD Therapy in DCM

Risk stratification for SCD in DCM is difficult and complex. Whilst SCD can be prevented by implantation of primary prevention ICDs, these devices come with their own risks.

ICD implantation can be associated with short and long-term complications including device infection or erosion, psychological impact and a risk of inappropriate shock. This also includes the risk of device malfunction with lead failure and the need for regular generator changes over the patient’s lifetime [82–84]. Given these complications from ICDs and that patients with DCM are often younger than their ischaemic counterparts, there is a need for better risk-stratification in DCM to identify those at elevated risk of SCD.

All RCTs for the prevention of SCD in DCM have focussed on LVEF as a stratifier of risk [2, 7]. Consequently, consensus guidelines for ICD implantation in DCM are limited to patients in New York Heart Association (NYHA) class II-III heart failure and with LVEF <35% [85, 86, 87••]. With respect to individual studies, there is some difference in the reported impact of ICD on all-cause mortality. In the DEFINITE trial, ICD implantation significantly reduced the risk of arrhythmic SCD but not all-cause mortality [2]. In contrast, the SCD-HeFT trial demonstrated that ICD implantation resulted in a reduction in all-cause mortality in a mixed cohort of patients with ischaemic and non-ischaemic causes of heart failure [7]. In a much quoted meta-analysis of five RCTs incorporating 1854 patients with DCM, there was a significant reduction in all-cause mortality with ICD implantation compared to OMT [5].

Most recently, The DANISH trial demonstrated that ICD implantation in DCM (76% idiopathic) was not associated with a significant reduction in all-cause mortality compared to OMT (21.6% vs 23.4%, respectively) in spite of a reduction in SCD rates [88••]. Possible explanations for this include a low SCD event rate (<5%) in the DANISH trial as well as excellent OMT with >90% of patients on ACE inhibitors / Beta-blockers and >50% on mineralocorticoid antagonists or with cardiac resynchronization therapy (CRT) [88••]. As yet, these findings have not resulted in a change in current practice guidelines for the use of ICD in DCM, but they do illustrate the need for more individualised risk stratification in DCM cohorts.

Risk stratification has been hampered by utilisation of heterogenous subsets of idiopathic DCM patients and a by a static risk assessment where predictions are based on a single time point with a lack of consideration of progression (deterioration or improvement) in the condition on OMT. The current focus is shifting towards better characterisation of the underlying aetiology of DCM and the development of multi-parametric risk-stratification models that incorporate time-dependent disease characteristics and novel biomarkers.
